# Gross Motor Performance, Participation and Quality of Life After Adapted Physical Activity Interventions in Pediatric Populations with Neuromotor Disability: A Systematic Review

**DOI:** 10.3390/children12070815

**Published:** 2025-06-21

**Authors:** Silvia Faccioli, Avola Marianna, Mangano Giulia Rita Agata, Sghedoni Silvia, Sassi Silvia

**Affiliations:** 1 Pediatric Rehabilitation Unit, AUSL IRCCS of Reggio Emilia, 42122 Reggio Emilia, Italy; silvia.sghedoni@ausl.re.it (S.S.); silvia.sassi@ausl.re.it (S.S.); 2 Physical Medicine and Rehabilitation Unit, Santa Marta and Santa Venera Hospital, 95024 Acireale, Italy; marianna.avola@aspct.it (A.M.); giulia.mangano@aspct.it (M.G.R.A.)

**Keywords:** motor activity, rehabilitation, exercise, sport, nervous system diseases, psychomotor performance, quality of life, activities of daily living

## Abstract

Background/Objectives: Physical activity is generally recommended, but youth with disabilities present increased sedentary behaviors. This is often due to limited or inaccessible exercise options. The aim of this systematic review was to report on the state of knowledge about the role of adapted physical activity (APA) in improving gross motor performance (query 1), participation and QoL (query 2) of children and adolescents with neurological motor disability. Methods: Pubmed, Scopus and Cinahl databases were enquired in October 2023 and updated in May 2025. Inclusion criteria were the following: any type of physical activity; pediatric subjects with any neuromotor disease; and any type of outcome measure regarding gross motor performance, participation or QoL. The risk of bias (RoB) was assessed by means of ROB 2, Robins-I and JBI tools. Results were synthetized focusing on the outcome measures and the type of activity proposed. Results: Thirteen and seven studies were included relative to queries 1 and 2, respectively. They all were RCTs, and some presented randomization RoB. Several types of APA (e.g., resistance, high-intensity circuit, running, cycling, aquatic and dance training) and of outcome measures were enquired, mostly focusing on subjects with cerebral palsy or Down syndrome. An increased time of moderate-to-vigorous physical activity, improvement in timed functional tests, muscle strength and stability were observed. Conclusions: APA may improve functioning, social participation and promote active lifestyle in pediatric persons with neuromotor disabilities, without adverse effects. In the future, more specific indications based on the functioning profile are advisable to orient professionals to define individualized safe training programs.

## 1. Introduction

Based on the World Health Organization (WHO) factsheet [1], physical activity is any bodily movement produced by skeletal muscles that requires energy expenditure and includes exercise, sports, active travel (cycling, walking), household chores and work-related physical activity. The WHO recommends regular, mostly aerobic, moderate- to vigorous-intensity physical activity across the week. This is often prevented in children or adolescents with a neurologic condition causing motor disability (i.e., cerebral palsy, acquired nervous system injury, hereditary spastic paraplegias, pediatric movement disorders or ataxias and any neurometabolic or neuromuscular diseases). These conditions may affect the child or adolescent gross motor function, i.e., gait performance, transferring, postural control and related activities. As a consequence, social participation, engagement in sports and physical activity are limited, with negative effects on quality of life [2,3,4].

Youth with disabilities are disproportionately affected by lower levels of physical activity and increased sedentary behaviors as a result of limited or inaccessible exercise options [2,3]. Participation in physical activities of children with physical disabilities is highly influenced by not only limitations in the activity (e.g., walking and mobility) and participation dimensions of ICF [4,5], but also by environmental and personal barriers [6]. Adapted settings appear to be necessary for children to develop physical and social skills that are invaluable for participating in their daily environment alongside peers [7]. As stated by Hutzler and Sherril, “Adapted physical activity (APA) science is research, theory and practice directed toward persons of all ages underserved by the general sport sciences, disadvantaged in resources, or lacking power to access equal physical activity opportunities and rights. APA services and supports are provided in all kinds of settings. Thus, research, theory and practice relate to the needs and rights in inclusive as well as separate APA programs” [8].

The distinction between exercise and physical activity is blurred. Exercise often refers to structured leisure-time physical activity such as participation in jogging and swimming, ‘‘keep-fit’’ activities, while physical activity may generally refer to “active lifestyle” [9]. Caspersen et al. (1985) [10] attempted to distinguish between physical activity and exercise. They defined physical activity as body movement produced by skeletal muscles; resulting energy expenditure varying from low to high; and positively correlated with physical fitness. Conversely, they attributed to exercise two additional characteristics: planned, structured, and repetitive bodily movement; and aimed at maintaining or improving physical fitness. Based on the American College of Sports Medicine’s guidelines, exercise is “any and all activity involving generation of force by the activated muscle (s) that results in disruption of a homeostatic state. In dynamic exercise, the muscle may perform shortening (concentric) contractions or be overcome by external resistance and perform lengthening (eccentric) contractions. When muscle force results in no movement, the contraction should be termed isometric” [11]. Winter et al. [9] concluded that the two terms may be interchangeable and will be used accordingly in the present study.

The health benefits of exercise in children with chronic physical illnesses and disabilities are starting to be thoroughly investigated. Carbone P.S. et al. reported that regular physical activity, recreation and sport participation improve the psychosocial well-being and quality of life of children with disabilities [3]. Willis C.E. et al. suggested that, in parallel to rehab programs, children participation should be enhanced with the use of adapted physical activity to improve activity-competences, self-efficacy and preferences for activities [6]. Morgan et al. reported benefits but also minor adverse events in non-ambulatory cerebral palsy adolescents [12]. Nonetheless, the role of APA and sports in the lives of children and adolescents with neuromotor disability is not yet well defined.

The aim of this systematic review was to report on the current knowledge regarding whether adapted physical activity and sport modify gross motor function, participation and/or quality of life in children and adolescents with neurological motor disability.

## 2. Materials and Methods

The present study consists of a systematic review of primary studies and was performed and reported in accordance with the reporting guidelines of the PRISMA statement [13] and Cochrane’s methodological recommendation [14]. The review protocol was registered on the PROSPERO public online register for systematic reviews (PROSPERO 2024 CRD42024518784) An amendment was made to the registered protocol, excluding case series.

The purpose of the systematic review was twofold and was structured according to the Patients, Intervention, Control and Outcome (PICO) framework for intervention.

Query 1 was: Does adapted physical activity improve mobility in children and adolescents with neurological motor disability? The query was refined using the following PICO:

- P: patients aged 2–18 with cerebral palsy, developmental disabilities, acquired nervous system injury, hereditary spastic paraplegias, pediatric movement disorders or ataxias, any neurometabolic or neuromuscular diseases, congenital, hereditary diseases and abnormalities.
- I: adapted physical activity.
- C: conventional therapy or no treatment.
- O: change in gross motor function, walking, postural control, postural balance, muscle strength, cardiorespiratory and muscular endurance.

Query 2 was: Does adapted physical activity improve participation and quality of life in children and adolescents with neurological motor disability? It was refined using the following PICO:

- P: patients aged 2–18 with cerebral palsy, developmental disabilities, acquired nervous system injury, hereditary spastic paraplegias, pediatric movement disorders or ataxias, any neurometabolic or neuromuscular diseases, congenital, hereditary diseases and abnormalities.
- I: adapted physical activity.
- C: conventional therapy or no treatment.
- O: change in quality of life and participation.

Search procedures are described in Supplementary Materials: Tables S1 and S2, concerning queries 1 and 2, respectively.

The literature search was performed on 19 October 2023 on several databases (Pubmed, Scopus, Cinahl) and updated on 30 May 2025.

The screening and selection of studies were performed by two independent authors (S.F., M.G.R.A., A.M.). Discrepancies between the two reviewers were resolved through discussion. Inclusion criteria included RCTs or Randomized Crossover Trials, age under 18 and English language. Studies performed in a healthcare setting or exclusively involving physiotherapy as intervention were excluded. Two independent authors reviewed the included articles (S.F., M.G.R.A., A.M.) and extracted data. Discrepancies between the reviewers were resolved through discussion. The results of every stage of selection were reviewed by the senior investigators (S.F. and S.S. (Sghedoni Silvia)). A third senior author (S.S. (Sassi Silvia)) was also involved in the discussion.

The following data were extracted by the included studies: study design, sample’s age, diagnosis and functional level by means of Gross Motor Function Classification System (GMFCS) whenever available, characteristics of experimental and control intervention, outcomes, and results for both queries. A descriptive analysis was performed, providing details of studies in summary tables and an analytical discussion of results. The diversity of interventions and outcome measures prevented from performing a meta-analysis.

The quality of studies was assessed by means of a checklist approach using the Joanna Briggs Institute (JBI) critical appraisal tools [15] for RCTs. The following thresholds were considered to define the overall quality: <65% YES as “critical”, 65–75% YES as “fair”, >75% YES as “good”. Limited relevance was attributed to blindness of participants and of those delivering treatment, because the type of treatment prevented the involved persons to be blind.

The risk of bias (RoB) was assessed also with a domain-based approach using version 2 of the Cochrane risk-of bias (ROB2) tool for RCTs [16]. The two independent groups of reviewers (S.F., A.M., M.G.R.A., and S.S. (Sghedoni Silvia)) assessed the methodological quality and the risk of bias of all the included studies. Any disagreement between the two groups was resolved through discussion among the authors. The assessment of quality and RoB did not provide criteria for excluding articles but for stratifying them.

No generative AI and AI-assisted technologies were used in the screening, selection and writing process.

## 3. Results

**Figure 1:**
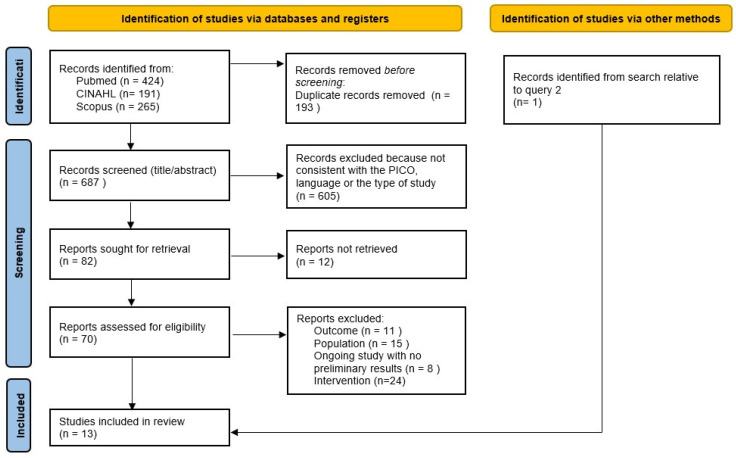
Prisma flow diagram relative to query 1.

**Figure 2:**
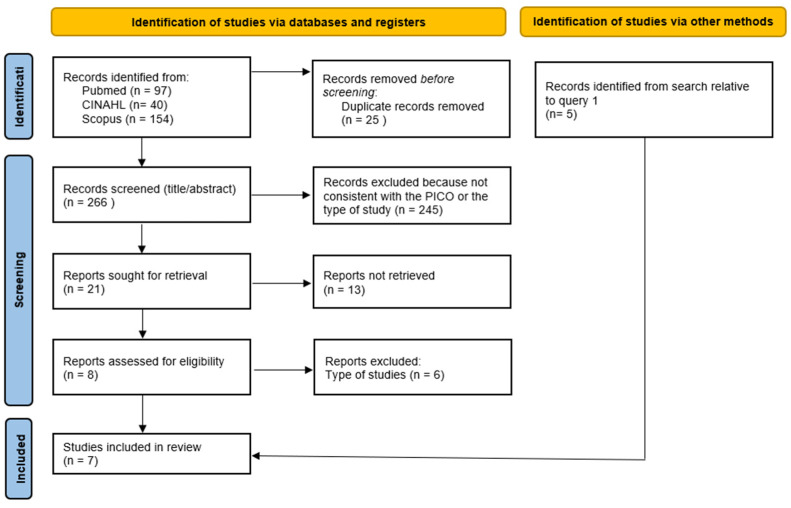
Prisma flow diagram relative to query 2.

Figure 1 and Figure 2 provide details (PRISMA flow diagram) about study identification and selection relative to queries 1 and 2, respectively. Finally, thirteen studies [17,18,19,20,21,22,23,24,25,26,27,28,29] were included for query 1 and seven [17,18,21,22,27,29,30] for query 2.

Characteristics of the studies, including the type of study, population, intervention, comparison, assessment program, outcome measures and results are shown in Table 1. Furthermore, a traffic light is included to represent the overall RoB for each individual study as assessed by means of the RoB2 tool [16]: green indicates low RoB and yellow some concerns. More details are reported in Supplementary Materials: Figure S1.

### 3.1. Quality of Studies and Risk of Bias

The quality assessment is represented in Supplementary Materials: Table S3. All RCTs reached a sufficient quality score based on criteria described in the Methods section. Lack of blindness to treatment assignment was a common feature across the included studies, but the type of intervention prevented patients and treatment providers to be blind. Therefore, we agreed on non-considering it as a significant quality limitation. Risk of bias assessment is represented through the “Traffic Light” tab in Figure S1 and Table 1 (first column). Relative to query 1, six RCTs [17,19,20,21,23,26] presented low risk of bias, with some concerns about the selection of the reported result in Elnaggar et al.’s study [23] and poor information about deviations from the intended intervention in Schranz et al.’s trial [17]. The other studies [18,22,24,25,27] presented some concerns relative to the lack of randomization process, poor information about deviations from the intended intervention, missing outcome data [27]. Regarding query 2, the RCT by Desmuth et al. [30] presented “some concerns” for not having clear information about deviations from the intended intervention.

### 3.2. Interventions Relative to Query 1

Several adapted physical activities were investigated, as different outcome measures were used to assess the results relative to functioning, participation and quality of life. The characteristics of the studies, including the outcome measure, the comparisons and results, are shown in Table 1.

Three studies enquired different types of progressive resistance training [17,24,25], but they were not comparable as they adopted different outcome measures. All the other studies focused on different kinds of adapted sports: adapted climbing [19], an ensemble of four different adapted activities (soccer, netball, T-ball and cricket) [22], Pilates-based exercises [20], aquatic activities [18,23], running [27], adapted cycling [21], gym-based APA combined with virtual reality [28] and dance [26]. All studies were RCTs. The control group performed nothing [28,30] or one of the following interventions: conventional therapy or neuromuscular training [18,19,20,22,23,24,25,26,27], a home-based high intensity circuit training [17], school-training [29] or a parent-led home program [21]. All three RCTs testing PRT [17,24,25] demonstrated a significant improvement of muscle strength compared to control groups. RCT evaluating aquatic exercises showed statistically significant improvement in functional assessment, such as walking distance for Declerck et al. [18] and sit-to-stand and stairs ability in the study by Elnaggar et al. [23]. The Sports Stars project, which included eight weekly one-hour sessions of combined sports-specific gross motor activity training with soccer, netball, T-ball and cricket, led to significant improvement in the modified Canadian Occupational Performance Measure (mCOPM) and the Test of Gross Motor Development (TGMD-2-total) [22]. Running training for 12 weeks improved significantly running abilities [27]. A measure of the amount of gross motor activity in daily living was assessed by means of actigraphs. An overall increase in active time was reported [21,28,29], with a significant improvement in terms of moderate-to-vigorous physical activity (MVPA) time involving the families in a remote collaboration-based PA [29].

### 3.3. Interventions Relative to Query 2

Six of the above-mentioned studies [17,18,21,22,27,29] enquired either functioning or participation and QoL. Therefore, they were included in both reviews. Only one study exclusively focused on the effects of APA on QoL in children with CP [30]. It investigated the effects of a stationary cycling intervention [30]: a general appreciation and adherence to the training and a within-group reduction in emotional and behavioral problems were reported, but without significant differences compared to controls (no treatment).

### 3.4. Outcome Measures

Several outcome measures were used. Nonetheless, they cannot be compared because of the heterogeneity of the samples, of the interventions and of the timing of the assessments. The most used outcome measure relative to query 1 was the Test of Gross Motor Development (TGMD) [22,26], a criterion-based test including 10–12 fundamental movement skills tasks in children at the age of 3–10 years; the Gross Motor Function Measure (GMFM) [24,25], a well-known tool assessing skills as rolling, crawling, sitting, standing, walking, running, climbing stairs and jumping; the Timed Up and Go Test (TUG) [17,22,28], a simple test enquiring both static and dynamic balance; the sit-to-stand test (STS) [25,26] related to the lower limb strength; and the Lateral Step Up test, assessing hip flexors strength [24,25].

The most used tool assessing participation and QoL (query 2) was the Pediatric Outcomes Data Collection Instrument (PODCI) [17,30], a questionnaire assessing functional health status in children with musculoskeletal disorders. Other reported assessment tools were: the Pediatric Quality of Life Inventory (PEDsQL) [30], a modular approach to measure health-related quality of life in children with different conditions, including multi-dimensional items (physical, emotional, social and school functioning); the participation section of the modified Canadian Occupational Performance Measure (m-COPM) [22], the Pediatric Evaluation of Disability Inventory-Computer Adapted (PEDI-CAT) [21], the Cerebral Palsy Quality of Life Questionnaire (CPQoL) [22], the Participation and Environment Measure for Children and Youth (PEM-CY) [27] and the Child Health Utility–9D (CHU9D) [21].

### 3.5. Side Effects

A few studies reported data regarding side effects [21,22] and declared no significant adverse events were recorded.

## 4. Discussion

The present SR included a group of RCTs focusing on APA to improve functioning or participation and quality of life of children with neuromotor disabilities. The studies are heterogeneous for the type of intervention and outcome measures. Therefore, it was not possible to compare the results by means of a meta-analysis. Nonetheless, some qualitative considerations may be shared.

Most of the studies involved children with cerebral palsy, which is in line with epidemiologic data, being the most frequent cause of pediatric disability in the world [31,32]. Furthermore, most samples included children or adolescents classified as GMFCS level I-II. Only a few studies included children at level III (mobility only possible with assistive devices as rollers or wheelchairs). Restricting participation to those walking with or without assistive devices mirrors the practical constraints faced by several children with disabilities while playing sports or another type of APA: the physical limitation is the major barrier, which might be overcome by adaptations of the context and/or assistive devices as facilitators. Moreover, many studies had a participant group drawn from convenience samples and included children, families and providers who were already involved in community or health facilities supplying physical activities or physiotherapy. This recruitment bias might have influenced the results.

Based on the present systematic review, PRT, at least three times per week for twelve weeks, improves muscle strength compared to conventional physical therapy (PT). Furthermore, PRT within a high intensity circuit training (HICT) was more effective than home-based PRT. Nonetheless, no change in gross motor performance was recorded after any type of PRT [17,24,25]. These data confirm findings from a previous Cochrane Review that enquired into the role of exercise in patients with CP [33].

Significant improvements in stability were obtained after 4-week Pilates-based training [20], after 12-week Aqua-PLYO (mixed exercise in waist-level immersion depth) [23] and 6-week Indian traditional dance [26] compared to conventional PT and assessed by means of BBT [20], of FSST [26] and of the dynamic limit of stability on force plates [23], respectively.

Studies enquiring swimming [18] and running [27] did not show their superiority compared to conventional PT at functional assessment, but goals were more successfully achieved specific to the proposed activity (WOTA) [18] or the individual subjects (GAS) [27]. Climbing appeared less effective in improving gait parameters compared to conventional physical therapy, in particular concerning knee extension in stance, and no overall statistically significant changes were reported by the authors [19].

The engagement of the family to promote PA significantly enhances the time of moderate to vigorous PA and reduces sedentary time in a cohort of adolescents with developmental disability [29].

Although sports might be considered more enjoyable and engaging than conventional PT, limited evidence was provided supporting their effectiveness in improving participation and QoL. Clutterbuck et al. [22] reported a significant improvement at mCOPM-Participation performance and satisfaction in the Sports Star group, maintained at follow-up, with no change in the waiting list group, but no significant differences at CPQoL between groups. A significant improvement in cycling group compared to PT for PedsQL psychosocial and emotional functioning sub scores was reported by Desmuth et al. [30], but no differences resulted at the total score. Gibson et al. [27] and Schranz et al. [17] reported that running training [27] and HICT [17], respectively, improved PEM-CY SCHOOL and PODCI Happiness sub scores, but again not the overall test score. Enjoyment must not be underrated as it is closely related to adherence, as confirmed by Declerck et al. [18], where the swimming intervention group reported enjoying the swimming sessions “very much” (Liekert 5), with a median 100% attendance rate. Relevant is the impact of peer-group activity as highlighted by Clutterbuck et al. [22] and Gibson et al. [27]. Group-based interventions involve reciprocal support, cognitive competences such as knowing game rules, persistence and confidence, and social competence, leading to a facilitation to the transition in community sports [22]. In general, we observe an improvement within experimental groups for participation and QoL outcome measures, without significant differences compared to conventional PT. This might be due to limited sample size in the included studies. Future research with larger samples might reinforce this trend. Furthermore, more specific outcome measures enquiring into children and adolescents’ satisfaction and engagement into the activity should be introduced. This aspect is still underrated.

In line with the literature, the authors decided to include physical therapy as comparison. Nonetheless, we think that APA and PT are not alternative but complementary. Physical therapy is undoubtedly essential during the developmental ages to promote the acquisition of gross and fine motor competences and again after interventions to counteract spasticity or dystonia or after functional neuro-orthopedic surgery [34,35,36,37]. Conversely, APA might be considered as “maintenance physical activity”, to counteract a decline in motor performances with age and promote fitness. This would be in line with the WHO recommendations [38] for children and adolescents with disability, and with the statement of the Italian care pathway for cerebral palsy, encouraging light intensity activities as fitness training (i.e., gross motor activity training, cycling, overground or treadmill walking, modified sports) integrated into the daily life of CP subjects [34]. Distinguishing the role of PT and APA may affect health policies, considering that physical therapy is delivered by therapists, while conversely APA is delivered by non-healthcare, but specifically trained physical activity professionals.

No indications about intensity and frequency may be drawn by the SR because of heterogeneity of studies. Therefore, at the moment the general recommendation by the WHO of starting with small amounts of physical activity, then gradually increase the frequency, intensity and duration over time, must be assumed [38].

No recurrent adverse events were reported in the examined studies. We may then assume that there are no major risks for children and adolescents with disability engaging in physical activity. At the moment, the evidence is too limited and does not permit to draw specific indications about which type of APA might be more or less effective based on the pathology or functioning profile. As the WHO recommendations state, it is necessary to refer to a healthcare professional to help determine the type and amount of activity individually appropriate for children and adolescents with disability [38].

### Limitations

The diversity of interventions and outcome measures across the included studies prevented to perform a meta-analysis. Only descriptive and general considerations may be drawn.

Our PICO focused on outcomes dealing with gross motor performances, but cardiorespiratory fitness is another relevant aspect involved in PA which should be assessed. Furthermore, limited attention was paid to flexibility among the included studies, mostly focusing on muscular (strength, power, endurance) or skill-related (e.g., balance, reaction time, speed) aspects.

## 5. Conclusions

There is limited evidence that APA may increase moderate-to-vigorous physical activity time and improve functioning (i.e., muscle strength, stability, timed functional tests) and social participation in children and youth with neuromotor disabilities, without adverse effects. Clinicians should encourage young patients with neuromotor disability to participate in adapted physical activity or sport, based on their preferences and individual characteristics.

Future research on APA should focus on the feasibility, adherence and engagement of children and adolescents to APA approaches, for example, by assessing the time spent in moderate-to-vigorous physical activity or reducing sedentary time over a long follow-up period, to verify the maintenance of the results and the real change in lifestyle.

Furthermore, studies with more homogeneous samples might permit drawing indications on appropriate types of exercise and the intensity and frequency to improve strength, flexibility and motor skills, providing specific outcome measures.

## Figures and Tables

**Table 1:** Characteristics of included studies relative to both queries 1 and 2.

Reference Study Design Overall RoB	Sample #	Intervention Group (IG)	Control Group (CG)	Assessment	Outcomes	Results Query 1 # ^§^	Results Query 2 # ^§^
Bohm H et al., 2014 [19] RCT crossover 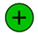	8 BSCP GMFCS I-II-III mean age 13.9 ± 4.3 (7–18) y	6 weeks of climbing therapy (CT), 1.5 h × 2/w; 4 w washout period between treatments	PT 1.5 h twice a week (NDT)	Pre- (T0) and post-intervention (T1)	*Query 1:* Walking speed, GPS, step length, step time	Mean T1-T0 Δ in: - Speed: in IG 0.09 ± 0.19, in CG 0.09 ± 0.16. - GPS: Δ in IG 0.04 ± 1.75, in CG 0.85 ± 2.66.	/
Clutterbuck G L et al., 2020 [22] RCT 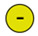	54 CP GMFCS I-II mean age IG 8.9 ± 2 and CG 8.6 ± 2 (6–12) y	Sports Stars: sports-specific gross motor activity training, sports education, teamwork development and confidence building for four sports: soccer, netball, T-ball and cricket; 8 weeks, 1 h × 1/w	Waiting list received standard care: PT mean 1.3 h (0–4 h) in 8 weeks.	Pre-intervention (T0), 8 w post intervention (T1), 12 w Fu (T2)	*Query 1*: mCOPM-activity, TGMD-2, GMFM-Challenge, TUG, MPST, 10 × 5mST, SBJ, Vertical Jump and Seated Throw. *Query 2*: m-COPM participation, CAPE-PAC, CP QOL-Child	Group by time effects using a linear mixed model analysis to compare groups: query 1 - mCOPM-Activity performance F 8.43 ** - mCOPM-Activity performance F 5.49 ** - TGMD-2-total F 5.19 ** - 10 × 5 mST F 3.84 * - MPST F 3.31 ** - SBJ F 2.81 * - GMFM-Challenge F 0.79 - TUG F 0.53 - Vertical jumping F 0.62 - Seated Throw F 0.09	Group by time effects using a linear mixed model analysis to compare groups: query 2: - mCOPM-Participation performance F 10.29 ** - mCOPM-Participation satisfaction F 7.21 ** - CAPE-PAC F 0.94 - CP QOL F 0.07–0.85 No adverse events: 2 trips (without injury).
Coman C et al., 2023 [20] RCT 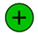	46 CP, GMFCS I-II, mean age IG 10y6m ± 2y6m and CG 10 y10 m ± 2y6m (7–17 y)	Pilates-based exercises with supplemental home exercise program, 60 m × 2/w for 4 w (480 m)	Conventional PT	Pre (T0) and 14 d post intervention (T1)	*Query 1:* 3DGA (speed, GPS, etc.), FWT, BBS, TSLS	Mean IG-CG Δ in T1-T0: - Speed 0.01 (0.09–0.07) - GPS 0.0 (0.7–0.7) - Percentage stance 1.4 (2.7–0.1) * - FWT 1.38 (2.18–0.56) ** - BBT 1.4 (2.22–0.58) ** - TSLS not normally distributed; no difference	/
Declerck M et al., 2016 [18] RCT 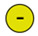	14 CP, GMFCS I-II-III mean age IG 8.7 ± 3.4, CG 11.8 ± 3.5 (7–17) y	10 w swimming program, 40–50 m × 2/w + conventional PT	Conventional PT	Pre (T0) and post (T1) intervention, after the 5 w Fu (T2)	*Query 1*: 1mFWT, WOTA 2, VAS, rFPS, PedsQL-MFS *Query 2*: 5-point Likert scale for enjoyment, % of adherence	Query 1: mean T1-T0 and T2-T1 Δ in: - 1mFWT IG 11.6 ± 18.2 and 18.9 ± 23.1; CG −8.3 ± 11.8 and 4.9 ± 17.5 - WOTA 2 IG 33.3 ± 8.7, 34.6 ± 9.3 versus ** CG 6.2 ± 9.3 and 3.7 ± 9.1	Query 2: - Median enjoyment IG 5 Median adherence IG 100%
Desmuth S K et al., 2012 [30] RCT 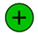	62 CP GMFCS I-II-III, median age IG 10.7 (8.5, 12.3), CG 11.2 (9.8, 13.3) range 7–18 y	Stationary cycling intervention, 1 h, 30 sessions over 12 w	No intervention	Pre (T0) and post intervention (T1)	*Query 2:* PedsQL, PODCI	/	Mean scores at T0 and T1 in: - PedsQL: IG T0 81.4 ± 2.2; T1 87.4 ± 12.3; CG T0 77.2 ± 11.6; T1 86.7 ± 14.6. - PODCI: IG T0 81.4 ± 2.2; T1 87.4 ± 12.3; CG T0 77.2 ± 11.6; T1 86.7 ± 14.6 Within-group improvements in IG PedsQL total score (+5.8) **, psychosocial health summary (+6.9) **, and school functioning (+8.0) **; PODCI satisfaction with symptoms on CG (−12.0) **
Elnaggar R K et al., 2022 [23] RCT 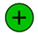	56 CP GMFCS I-II mean age IG 13.40 ± 1.37, CG 13.96 ± 1.45 (12–16) y	Aqua-PLYO (10′ low-impact aerobics warmup and cool-down and 25′ mixed exercises in waist-level immersion depth) 45 m × 3/w (2-day recovery in between) for 12 w	Conventional PT45 m × 3/w (non-consecutive days) for 12 w	Pre- (T0) and post-intervention (T1)	*Query 1:* Postural stability by LOSdynamic (M-DC, ReT, M-Vel, Mx-Exc, EP-Exc). Functional outcomes: 30s-STS, TUDS, DGI.	Mean scores at T0 and T1 in: - M-DC IG T0 74.32 ± 4.95, T1 80.36 ± 6.38 ** versus ** CG T0 73.11 ± 6.49, T1 75.32 ± 4.18 ** - ReT IG T0 0.66 ± 0.07, T1 0.59 ± 0.06 ** versus ** CG T0 0.68 ± 0.08, T1 0.65 ± 0.10 ** - M-Vel IG T0 4.53 ± 0.82, T1 5.14 ± 0.52 ** versus ** CG T0 4.21 ± 0.58, T1 4.52 ± 0.67 ** - Mx-Exc IG T0 71.18 ± 3.69, T1 77.04 ± 5.97 ** versus ** CG T0 69.36 ± 4.60, T1 72.14 ± 4.96 ** - EP-Exc IG T0 63.46 ± 3.99, T1 70.64 ± 5.42 ** versus ** CG T0 63.79 ± 3.68, T1 66.11 ± 4.41 ** - 30s-STS: IG T0 11.43 ± 2.23, T1 14.04 ± 1.90 ** versus ** CG T0 12.07 ± 1.68, T1 13.04 ± 1.50 ** - TUDS IG T0 14.39 ± 1.90; T1 12.3 4 ± 1.41 ** versus ** CG T0 14.12 ± 1.70, T1 13.24 ± 1.71 ** - DGI IG T0 18.36 ± 1.96, T1 22.18 ± 1.61 ** versus ** CG T0 17.61 ± 1.87, T1 19.93 ± 1.80 **	/
Gibson N et al., 2017 [27] RCT 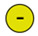	42 CP GMFCS I-II-III mean age IG 12.4 ± 2.7, CG 12.5 ± 2.8 (9–18) y	Running training at a community therapy organization 1 h × 2/w for 12 w and a home program × 2/w	Conventional PT	Pre (T0) and post intervention (T1)	*Query 1*: Improvement in running ability assessed by GAS and HLMAT; aerobic fitness by SRT, anaerobic fitness by MPST, agility by 10 × 5-Meter Sprint Test *Query 2*: PEM-CY	Mean Δ T1-T0 in: - GAS IG > CG 22.4 (16.2–28.5) ** - HILMAT IG > CG 0.8 (−2.7–4.3) - SRT IG > CG 1.0 (−0.2–2.2) - MPST IG < CG 6.8 (−40.6–27.0) - 10 × 5-Meter Sprint Test IG < CG −1.3 (−5.4–2.8)	PEM-CY School: IG > CG 1.2 (1.0–1.4) *
Hanssen B et al., 2022 [24] RCT 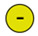	49 CP GMFCS I-II-III Mean age IG 8.3 ± 2.0, CG 8.5 ± 2.1 y.	Progressive resistance home training (PRT) × 3–4/w (non-consecutive days) for 12 w	Conventional PT	Pre- (T0) and post-intervention (T1)	*Query 1*: Isometric strength KE, KF, PF; LSU, STS, UHR, BHR, SLJ, 1MWT, GMFM	Mean Δ T1-T0 in: - Isometric strength KE IG 2.5 (0.8–4.2) versus * CG −0.4 (−2.1–1.2) - Isometric strength KF IG 6.0 (3.3–8.7) versus ** CG 0.8 (−1.9–3.4) - Isometric strength PF IG 3.6 (2.2–5.0) versus * CG 1.1 (−0.3–2.5) - LSU IG 2.7 (1.4–3.9) versus CG 1.1 (−0.2–2.3) - STS IG 2.7 (1.2–4.1) versus * CG 0.3 (−1.3–1.9) - UHR IG 9.1 (5.7–12.4) versus ** CG −1.5 (−4.7–1.7) - BHR IG 4.5 (1.9–7.1) versus CG 3.6 (1.1–6.0) - SLJ IG 5.8 (−0.2–11.8) versus CG 2.7 (−2.8–8.3) - 1MWT IG 5.6 (0.9–10.4) versus CG 3.6 (−1.0–8.1) - GMFM 0.7 (−0.6–2.1) IG versus CG 0.3 (−1.1–1.8)	/
Lee HK et al., 2024 [28] RCT 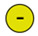	25 DD mean age 9 ± 1.6 y	VR + gym-based PA (5 m warm-up, 25 m VR PA, 25 m gym-based PA, 5 m cool-down) × 2/w for 12 w	No treatment	Pre- (T0), post-treatment (T1), 12 w Fu (T2)	*Query 1*: MVPA using GENEActive, SSRS	Mean Δ between groups at T1: - MVPA IG > CG 0.5 (−0.3–1.3) - SSRS IG > CG 11.2 (−1.1–23.4)	/
Raghupathy M K et al., 2021 [26] RCT 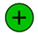	36 DS, mean age IG 7.8 ± 1.3, CG 8.4 ± 1.3 (6–10) y	Indian traditional dance 1 h × 3/w for 6 w	Neuromuscular training 1 h × 3/w for 6 w	Pre- (T0) and post-intervention (T1)	*Query 1*: TGMD-2, FSST, PBS	Mean Δ T1-T0 in: - GMQ-TGMD-2 IG 30.47 (24.5–36.49) versus ** CG 11.1 (8.35–13.77) - FSST IG 4.29 (3.76–4.83) versus ** CG 2.41 ( 2–2.82) - PBS IG 3.59 (2.93–4.25) versus CG 3.76 (3.1–4.33)	/
Scholtes V A et al., 2010 [25] RCT 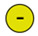	51 CP GMFCS I-II-III, mean age 10 y 5 mo	School PRT for lower limbs, 45–60 m × 3/w for 12 w	Conventional PT × 1–3/w	Pre- (T0), post-training (T1), 6 w Fu (T2).	*Query 1:* Mobility: GMFM-66, STS, LSU. 6-repetition maximum muscle strength on a leg-press. Isometric strength KE, KF, PF using a hand-held dynamometer. MobQues-28.	Mean T0, T1 and T3: - GMFM-66 IG T0 76.1 ± 12.8, T1 76.1 ±11.8, T3 76.6 ± 13.0; CG T0 71.8 ± 12.5, T1 73.1 ±12.4, T2 72.7 ± 12.8 - STS IG T0 12.9 ± 2.8; T1 13.3 ± 3.2; T2 14.3 ± 2.9; CG T0 10.8 ± 3.0; T1 12.1 ± 4.2; T2 12.7 ± 4.3) - LSU IG T0 15.6 ± 4.0; T1 17.0 ± 5.1; T2 17.5 ± 4.8; CG T0 13.3 ± 5.4; T1 15.4 ± 4.3; T2 15.8 ± 6.6) - Mob-Ques-28 IG T0 68.42 (20.93), T2 70.03 (23.49); CG T0 64.77 (26.26), T2 67.49 (20.33); IG T1 67.51 (24.58) versus * CG T1 66.43 (25.93) - Max strength IG T0 112.78 (21.28), T2 135.63 (31.87); CG T0 93.76 (20.18), T2 102.88 (26.76); IG T1 119.38 (26.61) versus * CG T1 100.80 (23.72) - Total isometric strength IG T0 18.04 (3.52), T1 19.88 (4.13), T2 20.39 (4.49); CG T0 15.94 (3.57), T1 16.65 (4.11), T2 17.80 (4.01)	/
Schranz C et al., 2018 [17] RCT 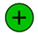	22 CP GMFCS I-II mean age IG 13.4 ± 2.4, CG 12.2 ± 2.7 y	HICT (as many repetitions as possible within 30 s intervals) × 3/w for 8 w	Home-based PRT × 3/w for 8 w	Pre- (T0), post-treatment (T1), 2 mo Fu (T2)	*Query 1*: Muscle strength (KF, MPST, TST, 6-MWT, GPS, TUG *Query 2*: ASKp, PODCI	Mean Δ T1-T0 in: - Muscle strength total IG 5.03 versus CG 1.03; KF IG 0.72 versus * CG 0.11; PF IG 1.68 versus * CG −0.39 - MPST IG 47.55 versus * CG 7.04 - TST IG −0.50 versus ** CG −1.83 - 6-MWT IG 5.6 versus CG 4.64 - GPS IG 0.65 versus CG 0.43 - TUG: IG −0.05 versus CG −1.26	Mean Δ T1-T0 in: - ASKp IG 2.44 versus CG 4.26 - PODCI IG 6.58 versus CG 2.81
Shen X et al., 2024 [29] RCT 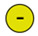	36 IDD, mean age 16.44 y ± 0.73	6-month school-based adapted PA 3 days a week + remote collaboration-based family PA	6-month school-based adapted PA 3 days a week	Pre- (T0), post-treatment (T1), 2 mo Fu (T2)	*Query 1*: Time spent at MVPA > 2800 CPM by means of actigraphs. Sedentary time at <100 CPM (LPA 100–2799 CPM). *Query 2*: WHOQOL-DIS-ID; PACES.	- Controls remained stable. Mean Δ T1-T0 and T2-T0: - Time at MVPA: IG 19.1 m at T1 and 5.64 m at T2, versus * CG. - Sedentary time: IG 56.34 m at T1 and 45.49 m at T2, versus * CG.	- Controls remained stable. Mean Δ T1-T0 and T2-T0: - WHOQOL-DIS-ID: IG 5.05 at T1 and −0.11 at T2 versus CG. - PACES: IG 7.12 at T1 and 4.44 at T2 versus * CG.
Toovey RAM et al., 2021 [21] RCT 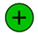	62 CP GMFCS I-II, mean age IG 9.11 ± 2.8, CG 9.1 ± 2.4 (6–15) y	Task-specific bicycleskills training program 2 h/d × 3 consecutive days at an outdoor park + 30 m/d home practice × 4 d, for 1 w	Parent-led bicycle skills home program 30–45 m/d, for 1 w	Pre-, 1 w post- (T1) and 3 mo post-intervention (T2)	*Query 1*: GAS at T1 and T1; PAQC; active time by means of Activ8 triaxial accelerometer *Query 2*: SPP-YC, SPP-C or SPP-A; CHU9D; PEDI-CAT	Mean Δ between IG and CG at T1 and T2 in: - GAS IG > CG T1 13.4 (8.5–18.2) **, T2 13.1 (5.4–20.7) ** - PAQC T2 0.3 (−0.3–0.9) - Active time T2 −0.3 (−2.0–1.5)	Mean Δ between IG and CG at T1 and T2 in: - SPP-global T1 0.2 (−0.8–0.7); T2 0.1 (−0.8–1.0) - CHU9D T1 0.02 (0.03–0.05); T2 0.01 (−0.05–0.06) - PEDI-CAT mobility T1 0.6 (−0.4–1.7); T2 0.3 (−1.2–1.8) - No adverse events

Legend: # age and results are reported in terms of mean values ±SD and (range) or median values and (IQR) or (1st, 3rd quartile), whenever available from original studies. § Statistically significant results are bolded, * *p* < 0.05, ** *p* < 0.01; Δ difference; 1mFWT, 1 min fast walk test; 1MWT, 1 min walk test; 6-MWT, 6 min walking test; 10 × 5 mST: 10 × 5 m sprint test; 30sec-STS, 30 s sit to stand test; 3DGA, 3D gait analysis; ASKp, activity scale for kids performance version; BBS, Berg balance scale; BHR, bilateral heel raise; BSCP, bilateral spastic cerebral palsy; CAPE-PAC, children’s assessment of participation and enjoyment-preferences of activities for children; CG, control group; CHU9D, Child Health Utility—9 domains; CI, confidence interval; COPM, Canadian Occupational Performance Measure; CP, cerebral palsy; CPM, counts per minute; CP QOL—cerebral palsy quality of life-child; CT, climbing therapy; d, day or days; DGI, dynamic gait index; DS, Down syndrome; FSST, four square step test; Fu, follow-up; FWT, functional walking test; GAS, goal attainment scale; GENEActive, Gravity Estimator of Normal Everyday Activity; GMAE2, gross motor ability estimator 2; GMFCS, Gross Motor Function Classification System; GMFM, Gross Motor Function Measure; GPS, gait profile score; h, hour or hours; HICT, high intensity circuit training; HLMAT, high-level mobility assessment tool; KE, knee extension; KF, knee flexion; IDD, intellectual and developmental disabilities; IG, intervention group; LOSdynamic, dynamic limits of stability (M-DC, movement directional control; ReT, reaction time; M-Vel, movement velocity; Mx-Exc, maximum excursion; EP-Exc, endpoint excursion); LPA, light physical activity; LSU, lateral step up; m, minutes; m, minutes; mCOPM, modified Canadian Occupational Performance Measure; MG, medial gastrocnemius; mo, months; MobQues-28, mobility questionnaire -28; MPST, muscle power sprint test; MVPA, moderate-to-vigorous physical activity; NDT, neurodevelopmental therapy; OR, odds ratio; PACES, physical activity enjoyment scale; PAQC, physical activity questionnaire for children; PBS, pediatric balance scale; PEDI-CAT, Pediatric Evaluation Of Disability Inventory-Computer Adapted; PedsQL, Pediatric Quality Of Life Inventory; PedsQL-MFS, multidimensional fatigue scale; PEM-CY, Participation And Environment Measure For Children And Youth; PF, plantar flexion; PODCI, Pediatric Outcomes Data Collection Instrument; PRT, progressive resistance training; PT, physical therapy; RCTs, randomized controlled trials; RF, rectus femoris; rFPS, revised faces pain scale; SBJ, standing broad jump; SLJ, standing long jump; SPP-YC, self-perception profile for young children; SPP-C, self-perception profile for children; SPP-A, self-perception profile for adolescents; SRT, 10 m shuttle run test; SSRS. Social Skills Rating System; ST, semitendinosus; STS, sit to stand; TGMD-2, Test Of Gross Motor Development (GMQ, gross motor quotient); TSLS, timed single leg stance test; TST, timed stairs test; TUDS, timed up and down stairs test; TUG, timed up and go test; UHR, unilateral heel raise; VAS, visual analogue scale; w, week or weeks; WHOQOL-DIS-ID, World Health Organization quality of life group-disabilities for persons with intellectual disability; WOTA, water orientation tests Alyn.

## Data Availability

The original contributions presented in this study are included in the article/Supplementary Materials. Further inquiries can be directed to the corresponding author.

## Supplementary Materials

The following supporting information can be downloaded at https://www.mdpi.com/article/10.3390/children12070815/s1. Table S1: Searching strategy relative to query 1; Table S2: Searching strategy relative to query 2; Table S3: Quality of studies relative to queries 1 and 2 assessed by means of the JBI; Figure S1: Risk of bias of included RCTs assessed by means of ROB 2.
