# Supplementary material for: Gross Motor Performance, Participation and Quality of Life After Adapted Physical Activity Interventions in Pediatric Populations with Neuromotor Disability: A Systematic Review
Source: Children (Basel). 2025 Jun 21;12(7):815. doi: 10.3390/children12070815 (PMC12293400; doi:10.3390/children12070815)
Supplement: Supplementary file 1 [file children-12-00815-s001.zip › Supplementary Material.docx]

Table S1. Searching strategy relative to query 1.

| **Database** | **Searching Strategy** | **n.** |
| --- | --- | --- |
| **Pubmed** | (("developmental disabilities"[MeSH Terms] OR "motor skills disorders"[MeSH Terms] OR "central nervous system diseases"[MeSH Terms] OR "congenital, hereditary, and neonatal diseases and abnormalities/abnormalities"[MeSH Terms] OR ("cerebral palsy"[Title/Abstract] OR "motor skill disorders"[Title/Abstract] OR "congenital disease"[Title/Abstract] OR "neonatal disease"[Title/Abstract] OR "hereditary disease"[Title/Abstract])) AND ((("acclimatization"[MeSH Terms] OR "acclimatization"[All Fields] OR "adaptation"[All Fields] OR "adaptations"[All Fields] OR "adapt"[All Fields] OR "adaptabilities"[All Fields] OR "adaptability"[All Fields] OR "adaptable"[All Fields] OR "adaptational"[All Fields] OR "adaptative"[All Fields] OR "adapte"[All Fields] OR "adapted"[All Fields] OR "adapting"[All Fields] OR "adaption"[All Fields] OR "adaptions"[All Fields] OR "adaptive"[All Fields] OR "adaptively"[All Fields] OR "adaptiveness"[All Fields] OR "adaptivity"[All Fields] OR "adapts"[All Fields]) AND ("exercise"[MeSH Terms] OR "exercise"[All Fields] OR ("physical"[All Fields] AND "activity"[All Fields]) OR "physical activity"[All Fields])) OR (("acclimatization"[MeSH Terms] OR "acclimatization"[All Fields] OR "adaptation"[All Fields] OR "adaptations"[All Fields] OR "adapt"[All Fields] OR "adaptabilities"[All Fields] OR "adaptability"[All Fields] OR "adaptable"[All Fields] OR "adaptational"[All Fields] OR "adaptative"[All Fields] OR "adapte"[All Fields] OR "adapted"[All Fields] OR "adapting"[All Fields] OR "adaption"[All Fields] OR "adaptions"[All Fields] OR "adaptive"[All Fields] OR "adaptively"[All Fields] OR "adaptiveness"[All Fields] OR "adaptivity"[All Fields] OR "adapts"[All Fields]) AND ("exercise"[MeSH Terms] OR "exercise"[All Fields] OR ("physical"[All Fields] AND "activities"[All Fields]) OR "physical activities"[All Fields])) OR "APA"[All Fields] OR "sports for persons with disabilities"[MeSH Terms] OR "sports"[MeSH Terms] OR "exercise"[MeSH Terms] OR ("adapted physical activities"[Title/Abstract] OR "adapted physical activity"[Title/Abstract] OR "APA"[Title/Abstract] OR "sport*"[Title/Abstract] OR "exercise*"[Title/Abstract])) AND ("walking"[MeSH Terms] OR "gait"[MeSH Terms] OR "postural balance"[MeSH Terms] OR "muscle strength"[MeSH Terms] OR "physical endurance"[MeSH Terms] OR "motor skills"[MeSH Terms] OR ("gross"[All Fields] AND ("motor"[All Fields] OR "motor s"[All Fields] OR "motoric"[All Fields] OR "motorically"[All Fields] OR "motorics"[All Fields] OR "motoring"[All Fields] OR "motorisation"[All Fields] OR "motorised"[All Fields] OR "motorization"[All Fields] OR "motorized"[All Fields] OR "motors"[All Fields]) AND ("functional"[All Fields] OR "functional s"[All Fields] OR "functionalities"[All Fields] OR "functionality"[All Fields] OR "functionalization"[All Fields] OR "functionalizations"[All Fields] OR "functionalize"[All Fields] OR "functionalized"[All Fields] OR "functionalizes"[All Fields] OR "functionalizing"[All Fields] OR "functionally"[All Fields] OR "functionals"[All Fields] OR "functioned"[All Fields] OR "functioning"[All Fields] OR "functionings"[All Fields] OR "functions"[All Fields] OR "physiology"[MeSH Subheading] OR "physiology"[All Fields] OR "function"[All Fields] OR "physiology"[MeSH Terms])) OR ("walk*"[Title/Abstract] OR "gait"[Title/Abstract] OR "postural balance"[Title/Abstract] OR "muscle strenght"[Title/Abstract] OR "physical endurance"[Title/Abstract] OR "motor skills"[Title/Abstract] OR "gross motor function"[Title/Abstract])) AND ("infant"[MeSH Terms] OR "child"[MeSH Terms] OR "adolescent"[MeSH Terms]) AND (("randomized controlled trial"[Publication Type] OR "controlled clinical trial"[Publication Type] OR "randomized"[Title/Abstract] OR "placebo"[Title/Abstract] OR "clinical trials as topic"[MeSH Terms:noexp] OR "randomly"[Title/Abstract] OR "trial"[Title]) NOT ("animals"[MeSH Terms] NOT "humans"[MeSH Terms]))) NOT ("editorial"[Publication Type] OR "letter"[Publication Type] OR "systematic review"[Publication Type] OR "meta-analysis"[Publication Type] OR "review"[Publication Type]) | **381 (10.19.2023)**  **+ 43 (05.30.2025)** |
| **Scopus** | (TITLE-ABS-KEY("adapted physical activit*" OR "sports for persons with disabilities" OR exercise)) AND (TITLE-ABS-KEY(walking OR gait OR "postural balance" OR "muscle strength" OR "physical endurance" OR "motor skills" OR "gross motor function")) AND (TITLE-ABS-KEY("cerebral palsy" OR "motor skill disorders" OR "central nervous system diseases")) AND ( LIMIT-TO (DOCTYPE,"ar" )) AND (LIMIT-TO (EXACTKEYWORD,"Randomized Controlled Trial") OR EXCLUDE ( EXACTKEYWORD,"Adult")) AND (LIMIT-TO (LANGUAGE,"English")) | **229 (10.19.2023)**  **+ 36 (05.30.2025)** |
| **CINAHL** | Filter: ALL CHILD ( cerebral palsy OR motor skill disorder OR developmental disabilities OR central nervous system disease ) AND ( adapted physical activity OR sport OR exercise ) AND ( Walking OR gait OR postural balance OR muscle strength OR physical endurance OR motor skills ) AND ( trial OR study) | **133 (10.19.2023)**  **+ 58 (05.30.2025)** |

Table S2. Searching strategy relative to query 2.

| **Database** | **Searching Strategy** | **n.** |
| --- | --- | --- |
| **Pubmed** | ((("developmental disabilities"[MeSH Terms] OR "motor skills disorders"[MeSH Terms] OR "central nervous system diseases"[MeSH Terms] OR "congenital, hereditary, and neonatal diseases and abnormalities/abnormalities"[MeSH Terms] OR ("cerebral palsy"[Title/Abstract] OR "motor skill disorders"[Title/Abstract] OR "congenital disease"[Title/Abstract] OR "neonatal disease"[Title/Abstract] OR "hereditary disease"[Title/Abstract])) AND ("sports for persons with disabilities"[MeSH Terms] OR "sports"[MeSH Terms] OR "exercise"[MeSH Terms] OR ((("acclimatization"[MeSH Terms] OR "acclimatization"[All Fields] OR "adaptation"[All Fields] OR "adaptations"[All Fields] OR "adapt"[All Fields] OR "adaptabilities"[All Fields] OR "adaptability"[All Fields] OR "adaptable"[All Fields] OR "adaptational"[All Fields] OR "adaptative"[All Fields] OR "adapte"[All Fields] OR "adapted"[All Fields] OR "adapting"[All Fields] OR "adaption"[All Fields] OR "adaptions"[All Fields] OR "adaptive"[All Fields] OR "adaptively"[All Fields] OR "adaptiveness"[All Fields] OR "adaptivity"[All Fields] OR "adapts"[All Fields]) AND ("exercise"[MeSH Terms] OR "exercise"[All Fields] OR ("physical"[All Fields] AND "activity"[All Fields]) OR "physical activity"[All Fields])) OR (("acclimatization"[MeSH Terms] OR "acclimatization"[All Fields] OR "adaptation"[All Fields] OR "adaptations"[All Fields] OR "adapt"[All Fields] OR "adaptabilities"[All Fields] OR "adaptability"[All Fields] OR "adaptable"[All Fields] OR "adaptational"[All Fields] OR "adaptative"[All Fields] OR "adapte"[All Fields] OR "adapted"[All Fields] OR "adapting"[All Fields] OR "adaption"[All Fields] OR "adaptions"[All Fields] OR "adaptive"[All Fields] OR "adaptively"[All Fields] OR "adaptiveness"[All Fields] OR "adaptivity"[All Fields] OR "adapts"[All Fields]) AND ("exercise"[MeSH Terms] OR "exercise"[All Fields] OR ("physical"[All Fields] AND "activities"[All Fields]) OR "physical activities"[All Fields])) OR "APA"[All Fields]) OR ("adapted physical activities"[Title/Abstract] OR "adapted physical activity"[Title/Abstract] OR "APA"[Title/Abstract] OR "sport*"[Title/Abstract] OR "exercise*"[Title/Abstract])) AND ("quality of life"[MeSH Terms] OR "community participation"[MeSH Terms] OR "social participation"[MeSH Terms] OR ("life quality"[Title/Abstract] OR "QoL"[Title/Abstract] OR "community participation"[Title/Abstract] OR "social participation"[Title/Abstract])) AND (("randomized controlled trial"[Publication Type] OR "controlled clinical trial"[Publication Type] OR "randomized"[Title/Abstract] OR "placebo"[Title/Abstract] OR "clinical trials as topic"[MeSH Terms:noexp] OR "randomly"[Title/Abstract] OR "trial"[Title]) NOT ("animals"[MeSH Terms] NOT "humans"[MeSH Terms]))) NOT ("editorial"[Publication Type] OR "letter"[Publication Type] OR "systematic review"[Publication Type] OR "meta-analysis"[Publication Type] OR "review"[Publication Type])) AND ((english[Filter] OR italian[Filter]) AND (allchild[Filter])) | **84 (10.19.2023)**  **+ 13 (05.30.2025)** |
| **Scopus** | ( TITLE-ABS-KEY ( "clinical trial*" OR "randomized controlled trial*" OR "rct*" OR "controlled clinical trial*" OR "random allocation" OR "randomly allocated" OR "allocated randomly" OR "double blind" OR "single blind" OR "cross over trial*" OR "placebo*" OR "factorial design" OR "factorial trial*" OR "intervention stud*" OR "quasi experimental" OR "clinical trials" OR "randomized controlled trial" OR "controlled clinical trial" OR "random allocation" OR "double-blind method" OR "single-blind method" OR "cross-over studies" OR "placebos" OR "multicenter study" ) ) AND ( TITLE-ABS-KEY ( "developmental disabilities" OR "cerebral palsy" OR "motor skill disorders" OR "central nervous system diseases" OR "neurological motor disabilit*" OR "neurodevelopmental disorder*" ) ) AND ( TITLE-ABS-KEY ( "community participation" OR "social participation" OR "life quality" OR "quality of life" OR "QoL" OR "participation" OR "social inclusion" OR "well-being" ) ) AND ( TITLE-ABS-KEY ( "adapted physical activit*" OR "adapted sport*" OR "sport*" OR "exercise" OR "physical activit*" OR "physical training" OR "rehabilitation" OR "therapy" ) ) AND ( TITLE-ABS-KEY ( "child*" OR "adolescent*" OR "pediatric*" OR "young" ) ) AND ( LIMIT-TO ( DOCTYPE , "ar" ) ) | **32 (10.19.2023)**  **+ 122 (05.30.2025)** |
| **CINAHL** | Filter: ALL CHILD ( cerebral palsy OR motor skill disorder OR developmental disabilities OR central nervous system disease ) AND ( adapted physical activity OR sport OR exercise ) AND ( community participation OR social participation OR life quality ) AND ( trial OR study ) | **29 (10.19.2023)**  **+ 11 (05.30.2025)** |

Table S3. Quality of studies relative to query 1 and 2 assessed by means of JBI.

| Reference | 1 | 2 | 3 | 4 | 5 | 6 | 7 | 8 | 9 | 10 | 11 | 12 | 13 | Overall | Judgment |
| --- | --- | --- | --- | --- | --- | --- | --- | --- | --- | --- | --- | --- | --- | --- | --- |
| Bohm et al. 2014 | Y | UN | Y | N | N | Y | Y | Y | Y | Y | Y | Y | Y | 10 | Good |
| Cluttebuck et al. 2020 | Y | Y | Y | N | UN | Y | Y | Y | Y | Y | Y | Y | Y | 11 | Good |
| Coman C et al, 2023 | Y | Y | Y | N | Y | Y | Y | Y | Y | Y | Y | Y | Y | 12 | Good |
| Declerck et al. 2016 | Y | UN | Y | N | N | Y | UN | Y | Y | Y | Y | Y | Y | 9 | Fair |
| Demuth S K et al, 2012 | Y | UN | Y | UN | UN | Y | Y | Y | Y | Y | Y | Y | Y | 10 | Good |
| Elnaggar et al. 2022 | Y | Y | Y | N | UN | Y | Y | Y | Y | Y | Y | Y | Y | 11 | Good |
| Gibson N et al, 2017 | Y | Y | Y | Y | UN | UN | Y | Y | Y | Y | Y | Y | Y | 11 | Good |
| Lee HK et al, 2023 RCT | Y | N | Y | N | N | N | Y | Y | Y | Y | Y | Y | Y | 9 | Fair |
| Hansenn et al., 2022 | Y | N | Y | N | N | N | Y | Y | Y | Y | Y | Y | Y | 9 | Fair |
| Raghupathy M K et al, 2021 | Y | Y | Y | Y | UN | UN | Y | Y | Y | Y | Y | Y | Y | 11 | Good |
| Shen X et al, 2024 | Y | UN | Y | N | N | UN | Y | Y | Y | Y | Y | Y | Y | 9 | Fair |
| Scholtes V A et al, 2010 | Y | Y | Y | Y | N | N | Y | Y | Y | Y | Y | Y | Y | 11 | Good |
| Schranz C et al, 2018 | Y | UN | Y | UN | UN | UN | Y | Y | Y | Y | Y | Y | Y | 9 | Fair |
| Toovey R A M et al, 2021 | Y | Y | Y | UN | N | Y | Y | Y | Y | Y | Y | Y | Y | 11 | Good |

Legend: 1. Was true randomization used for assignment of participants to treatment groups?; 2. Was allocation to groups concealed?; 3. Were treatment groups similar at the baseline?; 4. Were participants blind to treatment assignment?; 5. Were those delivering treatment blind to treatment assignment?; 6. Were outcomes assessors blind to treatment assignment?; 7. Were treatment groups treated identically other than the intervention of interest?; 8. Was follow up complete and if not, were differences between groups in terms of their follow up adequately described and analyzed?; 9. Were participants analyzed in the groups to which they were randomized?; 10. Were outcomes measured in the same way for treatment groups?; 11. Were outcomes measured in a reliable way?; 12. Was appropriate statistical analysis used?; 13. Was the trial design appropriate for the topic, and any deviations from the standard RCT design accounted for in the conduct and analysis?; Y, yes; N, no; U, unspecified.


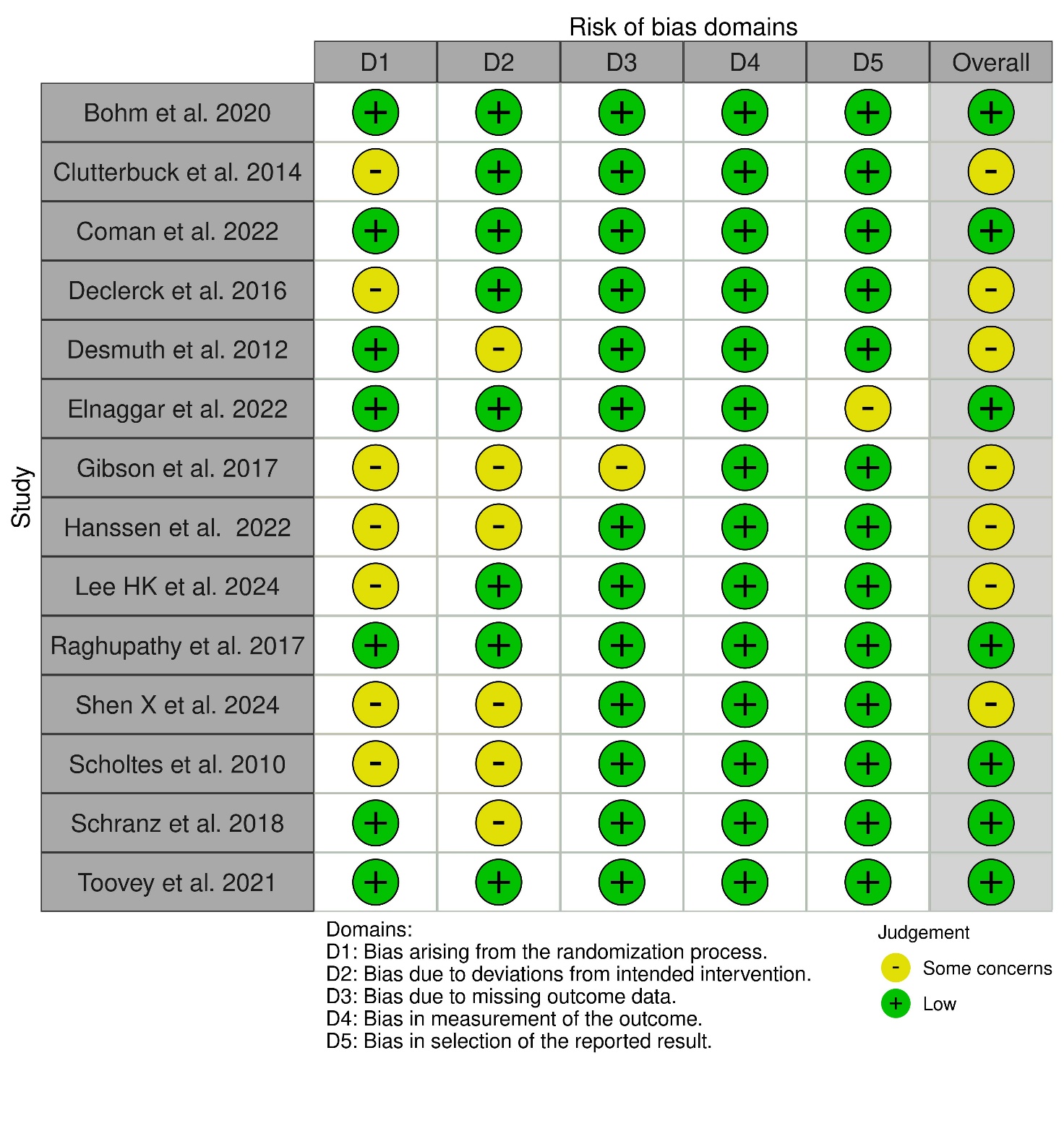


Figure S1. Risk of Bias of included RCTs assessed by means of ROB 2.
